# Correlation between Carbapenem Consumption and Carbapenems Susceptibility Profiles of *Acinetobacter baumannii* and *Pseudomonas aeruginosa* in an Academic Medical Center in Thailand

**DOI:** 10.3390/antibiotics11020143

**Published:** 2022-01-23

**Authors:** Taniya Paiboonvong, Phatchareeporn Tedtaisong, Preecha Montakantikul, Sarun Gorsanan, Woraphot Tantisiriwat

**Affiliations:** 1 Department of Pharmacy Practice, College of Pharmacy, Rangsit University, Pathum Thani 12000, Thailand; taniya.p@rsu.ac.th; 2 Department of Pharmacy, HRH Princess Maha Chakri Sirindhorn Medical Center, Faculty of Medicine, Srinakharinwirot University, Nakhon Nayok 26120, Thailand; phatchareeporn@g.swu.ac.th; 3 Division of Clinical Pharmacy, Department of Pharmacy, Faculty of Pharmacy, Mahidol University, Bangkok 10400, Thailand; preecha.mon@mahidol.ac.th; 4 Department of Social and Administrative Pharmacy, Faculty of Pharmaceutical Sciences, Huachiew Chalermprakiet University, Samut Prakarn 10540, Thailand; sarun103@yahoo.com; 5 Department of Preventive Medicine, HRH Princess Maha Chakri Sirindhorn Medical Center, Faculty of Medicine, Srinakharinwirot University, Nakhon Nayok 26120, Thailand

**Keywords:** carbapenem consumption, *A. baumannii*, *P. aeruginosa*, multidrug-resistant *A. baumannii*, multidrug-resistant *P. aeruginosa*

## Abstract

The emergent issue of carbapenem-resistant *Acinetobacter baumannii* (*A. baumannii*) and *Pseudomonas aeruginosa* (*P. aeruginosa*) is a major problem in Thailand. The wide use of carbapenems can increase selective pressure of bacterial resistance. The objective of this study was to determine the relationship between carbapenem consumption and the susceptibility rates of *A. baumannii* and *P. aeruginosa*, including multi-drug resistance (MDR) strains. This was a retrospective study. Carbapenem consumption and susceptibility profiles were collected from 2007 to 2013 at the Her Royal Highness Princess Maha Chakri Sirindhorn Medical Center, Thailand. We found that the susceptibility rate of *A. baumannii* to imipenem and meropenem from the sputum and the bronchoalveolar lavage (BAL) specimens was significantly decreased during the study period, but no significant change was found in the *P. aeruginosa* data. The relationship between carbapenem consumption and the susceptibility rate of *A. baumannii* had a clear association with the use of ertapenem. We found a statistically significant negative correlation between ertapenem consumption and the susceptibility rate of *A. baumannii* to imipenem (r = −0.91; *p* = 0.004) and meropenem (r = −0.97; *p* = 0.000) in the data from the non-ICU wards. In addition, imipenem use had a moderate negative correlation with the MDR *P. aeruginosa* data but no statistical significance (r = −0.714; *p* > 0.05). In conclusion, our study suggested there is an association between carbapenem use and the susceptibility of *A. baumannii* and *P. aeruginosa*. Notwithstanding this, information on ecological factors should be considered for further study. These findings showed the need to optimize the carbapenem prescription policy. Avoiding carbapenem overuse and rethinking the appropriate initial therapy might decrease the rate of resistant organisms.

## 1. Introduction

Antimicrobial resistance (AMR) has become a global public health concern for the last two decades. The multidrug-resistant Gram-negative bacteria (MDRGN), including carbapenem-resistant Gram-negative bacteria, poses a major problem related to increased hospital length of stay, healthcare costs and mortality rates [1,2,3]. The high prevalence of MDRGN has been reported in the Southeast Asian region, including Thailand, especially for *Acinetobacter baumannii* (*A. baumannii*) and *Pseudomonas aeruginosa* (*P. aeruginosa*). In this region, carbapenem–resistant *Acinetobacter baumannii* (CRAB) was the most common pathogen associated with nosocomial infections, followed by carbapenem-resistant *Pseudomonas aeruginosa* (CRPA), which are concerning in the face of difficult-to-treat infections [4,5,6,7]. In Thailand, the prevalence of Gram-negative nosocomial infections has been increasing since 2006, especially in the university hospital setting [8,9]. *A. baumannii* has caused nosocomial outbreaks with multidrug or carbapenem resistance, and it has rapidly increased in all regions of Thailand since 2000 [8,9,10,11,12,13]. Moreover, the rates of the multidrug-resistant *P. aeruginosa* (MDR-*P. aeruginosa*) with carbapenem resistance were found to be 71.65% among the tertiary hospitals across Thailand in last decade [14]. The overuse of antibiotics has been associated with the development of AMR, accelerated by selective pressure on the bacteria [15,16,17,18].

Carbapenems, broad-spectrum antibiotics, have been widely used for empirical treatment of nosocomial infections caused by Gram-negative bacteria [19,20]. They are usually reserved for the treatment of infections caused by MDRGN. Resistance to carbapenems in *A. baumannii* and *P. aeruginosa* could be explained by several mechanisms: carbapenemase, efflux pumps and decreased outer membrane permeability [19,20,21,22]. A correlation between carbapenem consumption and the rate of CRAB and CRPA has been described in several studies [23,24,25,26]. An antimicrobial stewardship program was an important tool to prevent and control AMR [27,28,29,30,31]. However, there are limited data on the impact of carbapenem consumption that focuses on CRAB and CRPA in Thailand. Therefore, this study aimed to determine the relationship between carbapenem consumption and the susceptibility of *A. baumannii* and *P. aeruginosa* in a tertiary care hospital.

## 2. Results

### 2.1. Carbapenem Consumption

**Figure 1:**
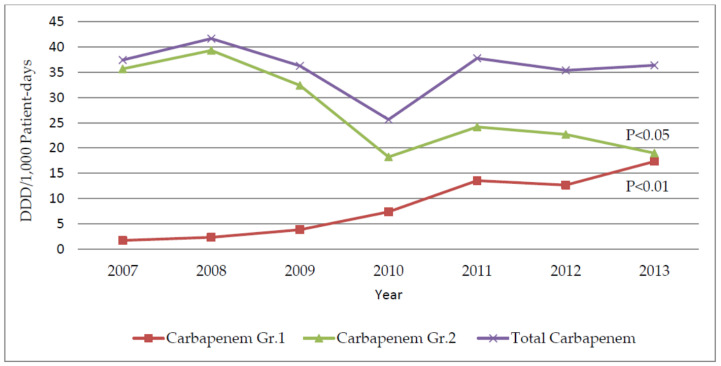
The annual data of the group 1 carbapenems, group 2 carbapenems and total carbapenems consumption.

The DDD/1000 patient-days of group 1 carbapenems (ertapenem) was significantly increased over time from 1.75 to 17.36 DDD per 1000 patient-days (r = 0.97; *p* = 0.000) after introducing the carbapenem control program (CCP). In contrast, the use of group 2 carbapenems was significant decreased from 2007–2013 (r = −0.84; *p* = 0.018). The carbapenem consumption intensities are presented in Figure 1 and Table 1.

### 2.2. Microbiology and the Susceptibility Profiles of A. baumannii and P. aeruginosa

A total of 1352 non-duplicated *A*. *baumannii* isolates and a total of 1386 non-duplicated *P*. *aeruginosa* isolates were collected during the study period. We found that the susceptibility rates of *A*. *baumannii* and *P*. *aeruginosa* to imipenem and meropenem were different among the specimens and the wards. The result from the blood specimens showed that the susceptibility rate of *A*. *baumannii* did not significantly change over time. However, the data from the sputum and the bronchoalveolar lavage (BAL) specimens showed that the susceptibility of *A*. *baumannii* to meropenem was significant for a negative correlation over time in the data from all wards (r = −0.83; *p* = 0.021). The data from the non-ICU wards showed significantly decreased susceptibility of *A*. *baumannii* to imipenem (r = −0.92; *p* = 0.003) and meropenem (r = −0.97; *p* = 0.000). However, no significant change was found for the susceptibility rate of *P*. *aeruginosa* in all specimens. These susceptibility profiles are shown in Table 2 and Table 3.

### 2.3. Relationship between Carbapenems Consumption and the Susceptibility Rate of A. baumannii to Imipenem and Meropenem and MDR-A. baumannii Data

**Figure 2:**
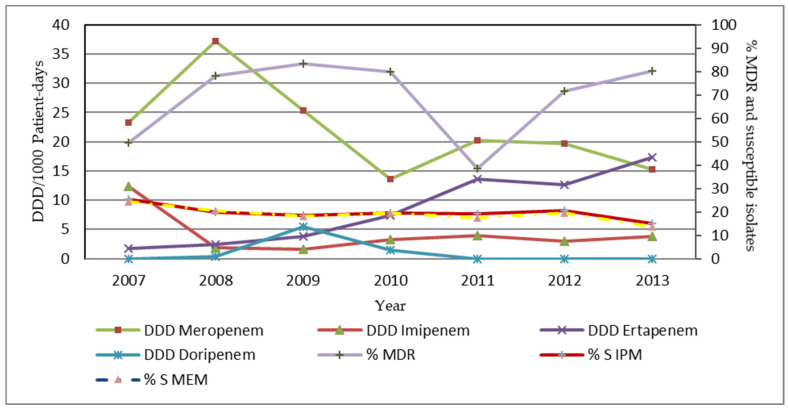
The annual consumption of the individual carbapenem, the percentage of susceptibility and the MDR-*A. baumannii* data from the sputum and the BAL specimens from all wards. %S IPM = the percentage of susceptibility to imipenem; %S MEM = the percentage of susceptibility to meropenem.

We found that the consumption of the group 1 carbapenems (ertapenem) had a negative correlation with the susceptibility rate of *A. baumannii* to imipenem and meropenem. There was a negative correlation between ertapenem consumption and the susceptibility rate of *A. baumannii* to meropenem (r = −0.79; *p* = 0.035) from the sputum and the BAL specimens from the data of all wards. Additionally, there was the strongest statistically significant negative correlation between ertapenem consumption and the susceptibility rate of *A. baumannii* to imipenem (r = −0.91; *p* = 0.004) and meropenem (r = −0.97; *p* = 0.000) in data from the non-ICU wards. The correlation is presented in Figure 2.

### 2.4. Relationship between Carbapenem Consumption and the Susceptibility Rate of P. aeruginosa to Imipenem and Meropenem and MDR-P. aeruginosa Data

**Figure 3:**
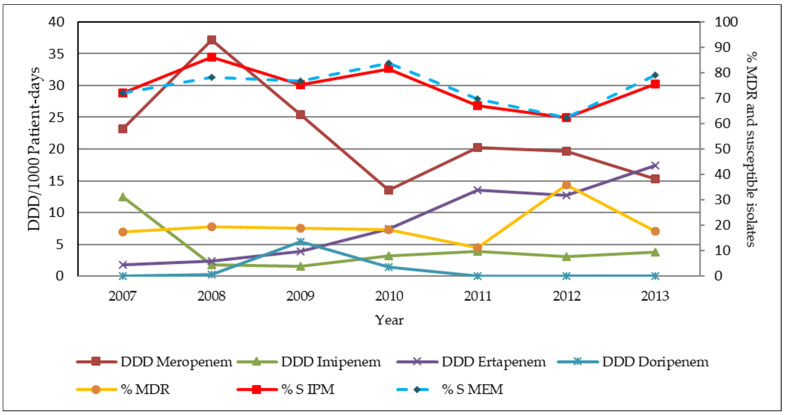
The annual consumption of the individual carbapenem, the percentage of susceptibility and the MDR-*P. aeruginosa* data from the sputum and the BAL specimens from the non-ICU wards. %S IPM = the percentage of susceptibility to imipenem. %S MEM = the percentage of susceptibility to meropenem.

The result from the sputum and BAL specimens of all wards demonstrated that the susceptibility of *P. Aeruginosa*, including the MDR strains, to imipenem and meropenem was slowly decreased during the study period. However, when the CCP was initiated into the MSMC system, it did not have a significant impact on the susceptibility of *P. aeruginosa*, including the MDR strains, to imipenem and meropenem. We found that carbapenem consumption did not show a statistically significant change in the susceptibility rate of *P. aeruginosa* to imipenem and meropenem. The data from the sputum and BAL specimens of the non-ICU wards showed that imipenem use had a moderate negative statistical correlation with MDR-*P*. *aeruginosa*. However, this correlation was not statistically significant (r = −0.71, *p* > 0.05). The correlation is presented in Figure 3.

## 3. Discussion

This study assessed the relationship between carbapenem consumption (Group 1 and Group 2) and the susceptibility patterns of the nosocomial infections caused by *A. baumannii* and *P. aeruginosa*. During the study period, Group 2 carbapenem consumption significantly decreased over time (r = −0.84, *p* = 0.018). Meropenem was the main carbapenem used, which was the most decreased carbapenem consumption among the group 2 carbapenems, especially in the period of the initiation of the CCP (2009–2010). On the other hand, ertapenem consumption significantly increased over time (r = 0.97, *p* = 0.000). Similarly, after replacement of the Group 2 carbapenems with ertapenem under the CCP, ertapenem consumption increased (*p* < 0.0001), while the group 2 carbapenem consumption significantly decreased over time (*p* = 0.028) [29].

The susceptibility rate of *A. baumannii* to imipenem and meropenem from the blood specimens decreased in the data from all wards (r = −0.39; *p* = 0.387 and r = −0.46; *p* = 0.294, respectively) and the ICU wards (r = −0.58; *p* = 0.175 and r = −0.63; *p* = 0.131, respectively) but showed no significant change during the years from 2007–2013. This finding was consistent with the result from Lee et al., which reported a significant decrease in the susceptibility rate of *A. baumannii* to imipenem and meropenem during their 7-year study period [29]. However, the increased susceptibility rate of *A. baumannii* was the most significant during the years from 2009–2010 (the period of the CCP initiation). Moreover, the data from the non-ICU wards showed the most increased susceptibility rates. While the highest increased susceptibility rate was identified in the year 2010, the MDR-*A*. *baumannii* rate was also increased. This situation could be from the outbreak of the MDR-*A*. *baumannii* in May and June 2010. According to the data from the sputum and the BAL specimens, the data from the non-ICU wards showed a statistically significant decrease in the susceptibility rate of *A. baumannii* to imipenem and meropenem over time. This finding could be explained by comparing the more severe patients in the ICU wards to the less severe patients in the non-ICU wards, leading to the higher carbapenem consumption and the higher resistance rate in the ICU wards.

The susceptibility rate of *P. aeruginosa* to imipenem and meropenem in all specimens from the data of all wards showed that the non-ICU wards and the ICU wards were slowly decreased during the study period. The susceptibility rates were increased the most during 2010, while the incidence rate of the MDR-*P. aeruginosa* was decreased the most after the initiation of the CCP. Moreover, as for the result from the blood isolates of the ICU wards, the rate of the susceptibility for *P. aeruginosa* was the highest in the year 2010, which was 100%, and the incidence rate of the MDR-*P. aeruginosa* was 0%. This data was only from two isolates; therefore, the incidence rate of the MDR-*P. aeruginosa* in the ICU wards might not represent for the trend of the MDR-*P. aeruginosa* in that year’s data.

Our study demonstrated that the ertapenem consumption had a significantly negative correlation with the susceptibility rate of *A. baumannii* to imipenem and meropenem. The data was obtained from the sputum and the BAL specimens from all wards and the non-ICU wards during the study period. Similar to the data from Lee et al., there were significantly negative correlations between the use of ertapenem and the susceptibility rate of *A. baumannii* to imipenem and meropenem [29]. However, the ertapenem use had no impact on the susceptibility rate of *A. baumannii* to imipenem, as reported by Sousa et al. [30]. In addition, there was no relationship between the proportion of CRAB isolates obtained from the infected patients and the intense use of ertapenem, as reported by Yoon et al. [31]. Therefore, the increased use of ertapenem might have selective pressure with the resistance to *A. baumannii*. In addition, CRAB could occur from multifactorial causes, including long-term use of broad-spectrum antimicrobials, consumption of antimicrobials, under/sub therapeutic dosage of the antimicrobials, prolonged stay in a hospital or long-term care facilities, ICU admission, underlying diseases, catheter indwelling and contamination by healthcare personnel [32,33,34]. Nevertheless, carbapenem consumption did not have a statistically significant correlation among *P. aeruginosa* data. We identified imipenem to have a statistically moderate negative correlation with MDR-*P*. *aeruginosa* but was statistically insignificant.

A study by Neves et al. showed that imipenem was independently related to the incidence of MDR strains (r = 0.67, *p* = 0.01) [35]. However, that study did not separate the data to each ward as in our study data. The reason might be explained by the imipenem resistance to *P. aeruginosa*, considered to be associated with a loss of the porin OprD combined with the activity of the chromosomal beta-lactamase (AmpC), while the overexpression of multidrug efflux pumps was considered to confer the meropenem resistance [36].

Our study had some limitations. First, we collected information on antimicrobial consumption, excluding information on ecological factors, which also influence AMR. It depicts association but not causal relations. More works need to be performed on finding different causation. Second, the exclusion of duplicated isolates may result in the under/overestimation of antimicrobial resistance. Finally, a lack of generalization may be concerning due to the single center being analyzed. Nevertheless, our findings are valuable in understanding the relationship of carbapenem consumption and the resistance for the implementation of a carbapenems stewardship program.

## 4. Materials and Methods

### 4.1. Study Design and Data Collection

This research was designed as a retrospective study at the Her Royal Highness Princess Maha Chakri Sirindhorn Medical Center (MSMC), which is a 360-bed university hospital with 12 medical wards and 5 intensive care units (ICU). There were 4 carbapenems, including imipenem, meropenem, doripenem and ertapenem in this study, which were divided into group 1 carbapenems (ertapenem) and group 2 carbapenems (imipenem, meropenem and doripenem). Antimicrobial consumption and the susceptibility pattern of *A*. *baumannii* and *P*. *aeruginosa* were analyzed. We collected the amounts of carbapenem consumption in grams from January 2007 to December 2013 from the MSMC database system described in yearly consumption. The amounts of the antimicrobial consumption were converted into “Define Daily Doses” (DDD). According to definitions from the Anatomical Therapeutic Chemical (ATC) Classification System and the World Health Organization (WHO), the DDD was expressed as days of therapy per 1000 patient days (DDD/1000 patient-days). The susceptibility rates were determined in all wards (17 wards), the non-ICU wards (12 medical wards) and the ICU wards (5 ICU wards), respectively.

Microbiological data and the susceptibility were collected from the database of the MSMC microbiological laboratory from January 2007 to December 2013. The susceptibilities were tested by disk diffusion, according to the Clinical and Laboratory Standards Institute (CLSI) standards during that period. The non-duplicated isolate was defined as “the first isolates of a species/patient/analysis period”. The research specified the period to exclude the duplicated isolates from the median patient-days in the nosocomial infected patients since the infections were identified until the discharge date. Therefore, the duplicated isolates were excluded from the study as of 18-day interval.

### 4.2. Statistical Analysis

The relationship between carbapenem consumption and the susceptibility were analyzed by using either the parametric Pearson’s or the non-parametric Spearman’s correlation coefficient. The *p* value of less than 0.05 was considered statistically significant. The Statistical Package for Social Science (SPSS) program, version 17, was used for all analyses.

## 5. Conclusions

We have identified an association between carbapenem use and the susceptibility of *A. baumannii* and *P. aeruginosa*. Our results indicate that carbapenem consumption is one of the contributing factors associated with the carbapenem-resistant rate. However, molecular analysis studies should be performed to elucidate the effect of carbapenem consumption on the susceptibilities of *A. baumannii* and *P. aeruginosa*. Avoiding carbapenem overuse and implementing an appropriate initial therapy might decrease the rate of resistant organisms. Further multicenter studies on other determinants that impact carbapenem resistance with more relevant data are needed.

## Figures and Tables

**Table 1:** The annual consumption of carbapenems (DDD/1000 patient-days) from 2007–2013.

	Carbapenems Consumption (DDD/1000 Patient-Days)						
Year	Meropenem	Imipenem	Doripenem	Ertapenem	Gr1	Gr2	Total
2007 ^#^	23.23	12.42	0	1.75	1.75	35.65	37.40
2008	37.19	1.81	0.29	2.35	2.35	39.29	41.64
2009	25.36	1.59	5.44	3.86	3.86	32.39	36.25
2010	13.56	3.21	1.47	7.39	7.39	18.24	25.63
2011	20.28	3.9	0	13.57	13.57	24.18	37.75
2012	19.67	3.02	0	12.67	12.67	22.69	35.36
2013	15.27	3.73	0	17.36	17.36	19.00	36.36
r	−0.63 a	−0.44 b	−0.23 b	0.97 a	0.97 a	−0.84 a	−0.22 a
*p* value	0.129	0.319	0.620	0.000 **	0.000 **	0.018 *	0.631

^#^ the carbapenem consumption was collected for 11 months; a, statistic calculation was based on the Pearson’s correlation coefficient; b, statistic calculation was based on the Spearman’s correlation coefficient; * Correlation is significant at the 0.05 level (2-tailed); ** Correlation is significant at the 0.01 level (2-tailed).

**Table 2:** The susceptibility rates of *A*. *baumannii* in different ward classification from 2007 to 2013 (*n* = 1352).

Isolates (*n*)	Wards	Antimicrobial Agents	Antimicrobials Susceptibility (%) by Year							Correlation	
			2007	2008	2009	2010	2011	2012	2013	r	*p*
Blood (154)	All wards (154)	Imipenem/cilastatin	42.86	36.36	50.00	57.89	32.00	33.33	34.38	−0.39	0.387
		Meropenem	42.86	36.36	52.38	57.89	32.00	33.33	30.00	−0.46	0.294
		MDR-AB	57.14	61.54	42.31	42.86	72.00	72.73	63.64	0.44	0.318
	Non-ICU wards (84)	Imipenem/cilastatin	28.57	60.00	50.00	84.62	35.29	50.00	50.00	0.13	0.788
		Meropenem	28.57	60.00	54.55	84.62	35.29	50.00	47.06	0.07	0.884
		MDR-AB	71.43	50.00	42.86	23.08	70.59	60.00	52.94	−0.04	0.940
	ICU wards (70)	Imipenem/cilastatin	57.14	16.67	50.00	0.00	25.00	16.67	14.29	−0.58	0.175
		Meropenem	57.14	16.67	50.00	0.00	25.00	16.67	7.69	−0.63	0.131
		MDR-AB	42.86	71.43	41.67	75.00	75.00	83.33	75.00	0.33	0.465
Sputum and BAL (1198)	All wards (1198)	Imipenem/cilastatin	25.25	20.00	18.40	19.55	19.31	20.43	14.90	−0.74	0.059
		Meropenem	24.49	20.69	18.01	19.55	17.59	19.46	13.56	−0.83 *	0.021
		MDR-AB	49.36	78.23	83.95	81.54	39.59	75.51	84.76	0.07	0.879
	Non-ICU wards (694)	Imipenem/cilastatin	24.53	22.22	23.30	21.62	20.61	19.19	15.18	−0.92 **	0.003
		Meropenem	25.00	23.33	22.33	21.62	18.60	18.18	13.68	−0.97 **	0.000
		MDR-AB	46.07	75.82	80.39	81.69	83.33	76.19	86.36	0.07	0.879
	ICU wards (504)	Imipenem/cilastatin	26.09	16.36	10.00	16.95	16.90	21.84	14.58	−0.25	0.589
		Meropenem	23.91	16.36	10.34	16.95	15.71	20.93	13.41	−0.29	0.528
		MDR-AB	53.73	82.14	90.00	81.36	84.51	74.73	83.00	−0.25	0.589

* Correlation is significant at the 0.05 level (2-tailed); ** Correlation is significant at the 0.01 level (2-tailed).

**Table 3:** The susceptibility rates of *P. aeruginosa* in different ward classification from 2007 to 2013 (*n* = 1386).

Isolates (*n*)	Wards	Antimicrobial Agents	Antimicrobials Susceptibility (%) by Year							Correlation	
			2007	2008	2009	2010	2011	2012	2013	r	*p*
Blood (98)	All wards (98)	Imipenem/cilastatin	100.00	83.33	53.85	77.78	61.54	58.33	78.57	−0.50	0.250
		Meropenem	100.00	83.33	53.85	77.78	53.85	58.33	71.43	−0.61	0.149
		MDR-PA	16.67	23.08	53.58	18.75	46.15	29.41	28.57	0.22	0.629
	Non-ICU wards (72)	Imipenem/cilastatin	100.00	88.89	54.55	75.00	63.64	77.78	50.00	−0.70	0.083
		Meropenem	100.00	88.89	54.55	75.00	63.64	77.78	50.00	−0.70	0.083
		MDR-PA	22.22	10.00	54.55	21.43	50.00	16.67	50.00	0.38	0.398
	ICU wards (26)	Imipenem/cilastatin	100.00	66.67	50.00	100.00	50.00	0.00	100.00	−0.28	0.549
		Meropenem	100.00	66.67	50.00	100.00	0.00	0.00	85.71	−0.39	0.383
		MDR-PA	0.00	66.67	50.00	0.00	33.33	60.00	12.50	0.02	0.965
Sputum and BAL (1288)	All wards (1288)	Imipenem/cilastatin	65.81	87.17	74.58	80.84	67.25	61.39	70.44	−0.39	0.394
		Meropenem	66.67	80.34	74.43	83.23	70.06	63.70	69.85	−0.30	0.507
		MDR-PA	22.49	17.39	21.02	18.24	14.20	34.35	23.03	0.34	0.450
	Non-ICU wards (930)	Imipenem/cilastatin	72.00	86.21	75.37	81.48	67.18	62.34	73.79	−0.49	0.270
		Meropenem	72.00	78.16	76.69	83.70	69.77	62.34	76.14	−0.28	0.548
		MDR-PA	17.86	20.00	19.55	19.69	12.30	33.62	18.49	0.07	0.879
	ICU wards (358)	Imipenem/cilastatin	54.76	90.00	72.09	78.13	67.50	61.05	64.29	−0.22	0.630
		Meropenem	57.14	86.67	67.44	81.25	71.05	63.16	58.33	−0.27	0.553
		MDR-PA	31.58	10.00	23.26	12.50	20.00	36.08	32.20	0.39	0.391

## Data Availability

The data that support the finding of this study are available on request from the corresponding author.

## References

[1] Pumart P., Phodha T., Thamlikitkul V., Riewpaiboon A., Prakongsai P., Limwattananon S. (2012). Health and economic impacts of antimicrobial resistance in Thailand. J. Health Syst. Res..

[2] Christian G.G., Dominique L.M., Otto C., Yehuda C. (2008). Clinical and Economic Impact of Common Multidrug-Resistant Gram-Negative Bacilli. Antimicrob. Agents Chemother..

[3] World Health Organization (2017). Guidelines for the Prevention and Control of Carbapenem-Resistant Enterobacteriaceae, Acinetobacter baumannii and Pseudomonas aeruginosa in Health Care Facilities.

[4] Molton J.S., Tambyah P.A., Ang B.S., Ling M.L., Fisher D.A. (2013). The global spread of healthcare-associated multidrug-resistant bacteria: A perspective from Asia. Clin. Infect. Dis..

[5] Mendes R.E., Mendoza M., Banga Singh K.K., Castanheira M., Bell J.M., Turnidge J.D., Lin S.S., Jones R.N. (2013). Regional resistance surveillance program results for 12 Asia-Pacific nations (2011). Antimicrob. Agents Chemother..

[6] Suwantarat N., Carroll K.C. (2016). Epidemiology and molecular characterization of multidrug-resistant Gram-negative bacteria in Southeast Asia. Antimicrob. Resist. Infect. Control.

[7] Kiratisin P., Chongthaleong A., Tan T.Y., Lagamayo E., Roberts S., Garcia J., Davies T. (2012). Comparative in vitro activity of carbapenems against major Gram-negative pathogens: Results of Asia-Pacific surveillance from the COMPACT II study. Int. J. Antimicrob. Agents.

[8] Danchaivijitr S., Judaeng T., Sripalakij S., Naksawas K., Plipat T. (2007). Prevalence of nosocomial infection in Thailand 2006. J. Med. Assoc. Thai..

[9] Werarak P., Waiwarawut J., Tharavichitkul P., Pothirat C., Rungruanghiranya S., Geater S.L., Chongthaleong A., Sittipunt C., Horsin P., Chalermskulrat W. (2012). *Acinetobacter baumannii* nosocomial pneumonia in tertiary care hospitals in Thailand. J. Med. Assoc. Thai..

[10] Dejsirilert S., Tiengrim S., Sawanpanyalert P., Aswapokee N., Malathum K. (2009). Antimicrobial resistance of *Acinetobacter baumannii*: Six years of National Antimicrobial Resistance Surveillance Thailand (NARST) surveillance. J. Med. Assoc. Thai..

[11] Hsu L.Y., Apisarnthanarak A., Khan E., Suwantarat N., Ghafur A., Tambyah P.A. (2017). Carbapenem-Resistant *Acinetobacter baumannii* and Enterobacteriaceae in South and Southeast Asia. Clin. Microbiol. Rev..

[12] Thirapanmethee K., Srisiri-a-nun T., Houngsaitong J., Montakantikul P., Khuntayaporn P., Chomnawang M.T. (2020). Prevalence of OXA-Type β-Lactamase Genes among Carbapenem-Resistant *Acinetobacter baumannii* Clinical Isolates in Thailand. Antibiotics.

[13] Paiboonvong T., Rodjun V., Houngsaitong J., Chomnawang M., Montakantikul P., Chulavatnatol S. (2020). Comparative in vitro activity of sitafloxacin against multidrug-resistant and carbapenem-resistant *Acinetobacter baumannii* clinical isolates in Thailand. Pharm. Sci. Asia.

[14] Khuntayaporn P., Montakantikul P., Mootsikapun P., Thamlikitkul V., Chomnawang M.T. (2012). Prevalence and genotypic relatedness of carbapenem resistance among multidrug-resistant P. aeruginosa in tertiary hospitals across Thailand. Ann. Clin. Microbiol. Antimicrob..

[15] Bell B.G., Schellevis F., Stobberingh E., Goossens H., Pringle M. (2014). A systematic review and meta-analysis of the effects of antibiotic consumption on antibiotic resistance. BMC Infect. Dis..

[16] Arepyeva M.A., Kolbin A.S., Sidorenko S.V., Lawson R., Kurylev A.A., Balykina Y.E., Mukhina N.V., Spiridonova A.A. (2017). A mathematical model for predicting the development of bacterial resistance based on the relationship between the level of antimicrobial resistance and the volume of antibiotic consumption. J. Glob. Antimicrob. Resist..

[17] Aremu T.O., Oluwole O.E., Adeyinka K.O. (2021). An understanding of the drivers of infectious diseases in the modern world can aid early control of future pandemics. Pharmacy.

[18] Ayukekbong J.A., Ntemgwa M., Atabe A.N. (2017). The threat of antimicrobial resistance in developing countries: Causes and control strategies. Antimicrob. Resist. Infect. Control.

[19] Papp-Wallace K.M., Endimiani A., Taracila M.A., Bonomo R.A. (2011). Carbapenems: Past, present, and future. Antimicrob. Agents Chemother..

[20] Codjoe F.S., Donkor E.S. (2017). Carbapenem Resistance: A Review. Med. Sci..

[21] Eichenberger E.M., Thaden J.T. (2019). Epidemiology and Mechanisms of Resistance of Extensively Drug Resistant Gram-Negative Bacteria. Antibiotics.

[22] Zavascki A.P., Carvalhaes C.G., Picao R.C., Gales A.C. (2010). Multidrug-resistant Pseudomonas aeruginosa and *Acinetobacter baumannii*: Resistance mechanisms and implications for therapy. Expert Rev. Anti. Infect. Ther..

[23] Mascarello M., Simonetti O., Knezevich A. (2017). Correlation between antibiotic consumption and resistance of bloodstream bacteria in a University Hospital in North Eastern Italy, 2008–2014. Infection.

[24] Cao J., Song W., Gu B., Mei Y.N., Tang J.P., Meng L., Yang C.Q., Wang H., Zhou H. (2013). Correlation between carbapenem consumption and antimicrobial resistance rates of *Acinetobacter baumannii* in a university-affiliated hospital in China. J. Clin. Pharmacol..

[25] Tan C.K., Tang H.J., Lai C.C., Chen Y.Y., Chang P.C., Liu W.L. (2015). Correlation between antibiotic consumption and carbapenem-resistant *Acinetobacter baumannii* causing health care associated infections at a hospital from 2005 to 2010. J. Microbiol. Immunol. Infect..

[26] Yang P., Chen Y., Jiang S., Shen P., Lu X., Xiao Y. (2018). Association between antibiotic consumption and the rate of carbapenem-resistant Gram-negative bacteria from China based on 153 tertiary hospitals data in 2014. Antimicrob. Resist. Infect. Control.

[27] Nicolau D.P., Carmeli Y., Crank C.W., Goff D.A., Graber C.J., Lima A.L., Goldstein E.J. (2012). Carbapenem stewardship: Does ertapenem affect Pseudomonas susceptibility to other carbapenems? A review of the evidence. Int. J. Antimicrob. Agents.

[28] Abdallah M., Badawi M., Amirah M.F., Rasheed A., Mady A.F., Alodat M., Alharthy A. (2017). Impact of carbapenem restriction on the antimicrobial susceptibility pattern of Pseudomonas aeruginosa isolates in the ICU. J. Antimicrob. Chemother..

[29] Lee C.M., Lai C.C., Wang Y.Y., Lee M.C., Hsueh P.R. (2013). Impact of susceptibility profiles of Gram-negative bacteria before and after the introduction of ertapenem at a medical center in northern Taiwan from 2004 to 2010. Diagn. Microbiol. Infect Dis..

[30] Sousa D., Castelo-Corral L., Gutiérrez-Urbón J.M., Molina F., López-Calviño B., Bou G., Llinares P. (2013). Impact of ertapenem use on Pseudomonas aeruginosa and *Acinetobacter baumannii* imipenem susceptibility rates: Collateral damage or positive effect on hospital ecology?. J. Antimicrob. Chemother..

[31] Yoon Y.K., Yang K.S., Lee S.E., Kim H.J., Sohn J.W., Kim M.J. (2014). Effects of Group 1 versus Group 2 carbapenems on the susceptibility of *Acinetobacter baumannii* to carbapenems: A before and after intervention study of carbapenem-use stewardship. PLoS ONE.

[32] Le Hello S., Falcot V., Lacassin F., Mikulski M., Baumann F. (2010). Risk factors for carbapenem-resistant *Acinetobacter baumannii* infections at a tertiary care hospital in New Caledonia. Scand. J. Infect. Dis..

[33] Sheng W.H., Liao C.H., Lauderdale T.L., Ko W.C., Chen Y.S., Liu J.W., Lau Y.J., Wang L.H., Liu K.S., Tsai T.Y. (2010). A multicenter study of risk factors and outcome of hospitalized patients with infections due to carbapenem-resistant *Acinetobacter baumannii*. Int. J. Infect. Dis..

[34] Zheng Y.L., Wan Y.F., Zhou L.Y., Ye M.L., Liu S., Xu C.Q., He Y.Q., Chen J.H. (2013). Risk factors and mortality of patients with nosocomial carbapenem-resistant *Acinetobacter baumannii* pneumonia. Am. J. Infect. Control.

[35] Neves M.T.d, Lorenzo M.E.P.d, Almeida R.A.M.B., Fortaleza C.M.C.B. (2010). Antimicrobial use and incidence of multidrug-resistant Pseudomonas aeruginosa in a teaching hospital: An ecological approach. Rev. Soc. Bras. Med. Trop..

[36] El Amin N., Giske C.G., Jalal S., Keijser B., Kronvall G., Wretlind B. (2005). Carbapenem resistance mechanisms in Pseudomonas aeruginosa: Alterations of porin OprD and efflux proteins do not fully explain resistance patterns observed in clinical isolates. APMIS.

